# Intermittent hypoxic stimulation promotes efficient expression of Hypoxia-inducible factor-1α and exerts a chondroprotective effect in an animal osteoarthritis model

**DOI:** 10.1371/journal.pone.0319976

**Published:** 2025-04-01

**Authors:** Ryota Cha, Shuji Nakagawa, Yuji Arai, Atsuo Inoue, Naoki Okubo, Yuta Fujii, Kenta Kaihara, Kei Nakamura, Tsunao Kishida, Osam Mazda, Kenji Takahashi

**Affiliations:** 1 Department of Orthopaedics, Graduate School of Medical Science, Kyoto Prefectural University of Medicine, Kyoto, Japan; 2 Department of Sports and Para-Sports Medicine, Graduate School of Medical Science, Kyoto Prefectural University of Medicine, Kyoto, Japan; 3 Department of Immunology, Graduate School of Medical Science, Kyoto Prefectural University of Medicine, Kyoto, Japan; University of Vermont College of Medicine, UNITED STATES OF AMERICA

## Abstract

Hypoxia-inducible factor-1α plays an important role in the homeostasis of articular cartilage in hypoxic environments. Therefore, modulation of hypoxia-inducible factor-1α by regulating the oxygen environment could be a useful treatment for osteoarthritis. This study aimed to assess the chondroprotective effects of intermittent hypoxia on cultured chondrocytes and an animal model of osteoarthritis. *In vitro*, human chondrocytes were exposed to 2 h of hypoxic stimulation three times at 1-h intervals, and protein and gene expression of hypoxia-inducible factor-1α, ACAN, and cell viability was measured over time. *In vivo*, 8-week-old male Wistar rats were injected with monosodium iodoacetate to induce osteoarthritis and then reared in 12% hypoxia for 24 h, followed by 24 h in steady oxygen, repeated alternately for a total of 28 days. A histological analysis was performed on days 8 and 28. In the intermittent hypoxia group, each protein expression increased with each repeated hypoxic stimulation to human chondrocytes; finally, the protein level was significantly higher with intermittent hypoxia than with continuous hypoxic stimulation, cell viability was increased, and gene expression was not significantly increased. In the osteoarthritis animal model, for 8 days, there were stronger hypoxia-inducible factor-1α staining and no significant differences in articular cartilage destruction. Furthermore, for 28 days, there was significantly less articular cartilage destruction in the rat osteoarthritis model with intermittent hypoxia than with steady oxygen rearing. Intermittent hypoxia increased cartilage metabolism by increasing hypoxia-inducible factor-1α proteins in articular chondrocytes, which may be effective in preventing articular cartilage degeneration in a rat osteoarthritis model.

## Introduction

Osteoarthritis (OA) is the most common cause of joint disease and is caused by degeneration of the articular cartilage [1]. An estimated 240 million people worldwide have OA [2], and this number is expected to increase due to longer life expectancy [3]. OA is a disease affecting all structures of the joint and is characterized by the destruction of articular cartilage and changes in other joint components such as bone, synovium, and muscle [4]. These result in the main clinical manifestations of OA, including pain, limited range of motion, and joint swelling. Reduced joint function leads to reduced activities of daily living (ADL) [5] and is a major cause of work disability in the elderly [6]. Surgical treatments such as joint replacement are often the treatment of choice for advanced OA, and the lost wages and increased healthcare costs due to work disability related to OA are a significant burden [7]. However, the mechanisms underlying the progression of cartilage degeneration due to OA are not fully understood [8]. Therefore, no effective treatment has been established to completely inhibit the progression of OA [9,10], and new treatment methods are required.

Oxygen and nutrients are carried primarily by the bloodstream and essential for the survival of living cells and organs. However, in the body there are non-vascular tissues, such as the cornea and intervertebral discs, of which articular cartilage is a prime example [11,12]. Articular cartilage receives nutrients and oxygen supply from the joint fluid to maintain homeostasis but is subjected to a hypoxic environment and has a superior hypoxic stress response compared to that of blood flow-rich tissues [13,14]. The oxygen environment in joint fluid is normally maintained at 6–9%, with oxygen levels decreasing to approximately 1% in articular cartilage [15].

Hypoxia-inducible factor (HIF)-1α plays an important role in adapting to this markedly hypoxic environment. HIF-1α is hydroxylated and modified at proline residues in the oxygen-dependent degradation domain, where it acts as a sensor of oxygen concentration by prolyl hydroxylase domain protein (PHD) under steady-state oxygen conditions. Subsequently, the tumor suppressor molecule, pVHL, binds and is ubiquitinated by ubiquitin ligase complex and degraded by the proteasome. However, in hypoxic conditions, this hydroxylation modification is inhibited, and the HIF-1α protein in chondrocytes accumulates and stabilizes. Subsequently, this forms dimers with HIF-β and activates transcription of anabolic factors such as Sox 9, Aggrecan, and type II collagen, maintaining articular cartilage homeostasis [16–18]. Thus, HIF-1α plays an important role in articular cartilage homeostasis, HIF-1α-deficient chondrocytes are unable to maintain adenosine triphosphate (ATP) levels in a hypoxic microenvironment, and vascular endothelial growth factor, which is the main angiogenic target of HIF-1α, and Aggrecan levels are greatly reduced [19]. Furthermore, in OA, as the articular cartilage degenerates, oxygen levels in the tissue increase, PHD-induced degradation of HIF-1α is accelerated, the protein level of HIF-1α in the cells decreases, and the expression of HIF-1α-responsive genes is reduced [20]. If HIF-1α can accumulate and is stabilized in OA chondrocytes by controlling oxygen levels in the articular cartilage tissue, the progression of cartilage degeneration can be prevented, or cartilage can be regenerated.

Hypoxia is “chronic” or “sustained” when experienced continuously by the cells and “acute” or “intermittent” when fluctuating; the cells behave differently under each of these environments [21,22]. Regarding the relationship between hypoxia duration and intracellular HIF-1α protein levels, the amount of intracellular HIF-1α protein increases after a short period of hypoxia from steady-state oxygen (acute); however, after a prolonged period of hypoxia (sustained), PHD is activated by negative feedback and intracellular HIF-1α protein levels decrease [23]. Conversely, studies in tumor and vascular endothelial cells have reported that repeated acute hypoxia (intermittent) results in more efficient gene expression and reduced degradation of HIF-1α than sustained hypoxia [24–26]. We have also previously successfully regulated HIF-1α expression in synovial cells and rheumatoid arthritis (RA) animal models using a hypoxic environment [23,27]. We hypothesized that intermittent hypoxia (IH) in the articular cartilage could efficiently increase HIF-1α and have a therapeutic effect on OA. We, therefore, aimed to evaluate the chondroprotective effects of IH on cultured chondrocytes and an animal OA model and to develop novel treatments for OA using this hypoxic environment.

## Materials and methods

### Preparation of human chondrocytes (HCHs)

HCHs (C-12710) purchased from Promocell were used as chondrocytes (PromoCell, Heidelberg, Germany) and were isolated from healthy 86- and 54-year-old male Caucasian articular cartilages (lot numbers 475Z011.1 and 479Z003.2, respectively) of the hip joints. HCHs were monolayered in cultures in 75 cm^2^ flasks (Corning Inc., Corning, NY) in chondrocyte growth medium (C-27101) (Nacalai Tesque, Kyoto, Japan) containing 1% penicillin-streptomycin mixed solution (complete medium) (PromoCell GmbH, Heidelberg, Germany) and penicillin-streptomycin mixed solution (Nacalai Tesque, Kyoto, Japan) at 37°C in 5% CO_2_/95% humidified air. The culture medium was changed on the day after seeding and every 2–3 days thereafter.

### Cultures in hypoxia

Cultured HCHs were treated with trypsin/ethylenediaminetetraacetic acid (Nacalai Tesque, Kyoto, Japan), resuspended in complete medium, and seeded onto a 35-mm dish (AGC Techno Glass, Shizuoka, Japan) at a density of 1 ×  10^5^ cells per well and 96-well plate (FALCON, Kanagawa, Japan) at a density of 1 ×  10^4^ cells per well. When the cells reached 70–80% confluence, incubation was started under IH or continuous hypoxia. Previous studies defined hypoxia as an oxygen concentration of 1% [23,27–30], and our experiments were conducted accordingly. After 2 h of incubation under hypoxic conditions of 1% O_2_, 5% CO_2_, and 94% N_2_ using a multi-gas incubator (9000EX, Wakenyaku Co. Ltd., Kyoto, Japan) as a hypoxic environment device, the cells were moved into a CO_2_ incubator (Forma 310 Direct Heat CO_2_ Incubators, Thermo Scientific, Waltham, MA) for 1 h under steady oxygen conditions of 21% O_2_, 5% CO_2_, and 74% N_2_. This was repeated three times as one cycle for the IH group. In the intermittent group, a total of 2 h of steady oxygen incubation and 6 h of hypoxia incubation were performed. In the SH group, the aforementioned 2 h of incubation under steady oxygen followed by 6 h of incubation under hypoxic conditions was performed. The incubator used for hypoxic conditions took an average of 45 minutes to reach the 1% O2 target after the transfer of cultured cells, and the oxygen concentration during incubation was constantly monitored to ensure that the oxygen concentration was as planned.

### Western blot analysis

**Fig 1:**
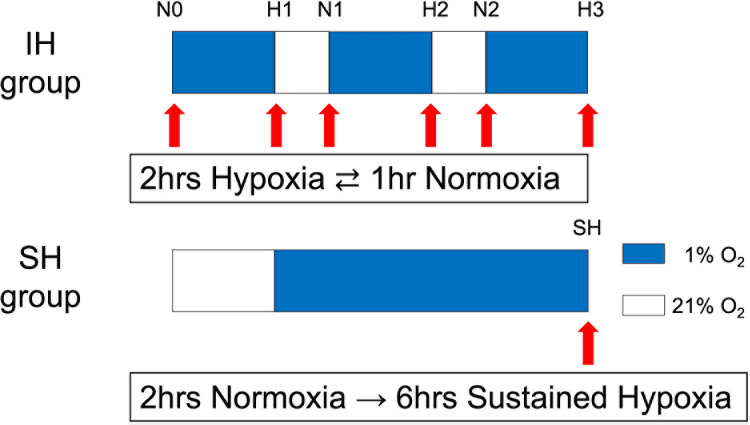
HCHs were incubated in an intermittent hypoxic environment (1% hypoxia, 2 h; 21% steady-state oxygen, 1 h). Proteins were collected at the end of the hypoxic cycle (H1, H2, H3) and the end of the steady oxygen cycle (R0, R1, R2). In the SH culture, hypoxia proteins were recovered after 6 h of continuous incubation in a hypoxic environment; hence, the cumulative hypoxic exposure time was identical to that of H3.

In the IH group, proteins were collected at the timing of the change in the oxygen environment, 0, 2, 3, 5, 6, and 8 h after the start of culture (N0, H1, N1, H2, N2, H3) and, in the SH group, proteins were collected 8 h after the start of culture (Fig 1).

The cells were placed in a 35-mm dish and washed twice with phosphate-buffered saline (PBS); overall, 150 µl of RIPA Buffer (Nacalai Tesque, Kyoto, Japan) was used to scrape and collect the cells. The cells were then centrifuged at 15,000 rpm for 5 min, and the supernatant was aspirated. Protein concentrations were estimated by bicinchoninic acid assay, and equal amounts of total protein were loaded onto gels and underwent sodium dodecyl sulfate polyacrylamide gel electrophoresis (SDS-PAGE). Samples containing 4.25 µg of protein were separated by electrophoresis on 10% Bis-Tris Gel NuPAGE® using 5% MOPS SDS Running Buffer (Thermo Fisher Scientific, Inc., Waltham, MA). Separated proteins were wet blotted onto a PVDF membrane, the membrane was shaken, and blocked in Blocking One solution (Nacalai Tesque, Kyoto, Japan) at 20°C for 30 min, incubated overnight at 4°C in a solution containing a 1:1,000 dilution of the primary antibody (HIF-1α [ab51608, Abcam, Cambridge, UK], SOX9 [ab185966, Abcam], and Aggrecan [ab3778, Abcam]; β-actin (A5316, Sigma-Aldrich, St. Louis, MO) was used as a loading control. The cells were then washed 2–3 times with tris-buffered saline with Tween 20 (TBST) and incubated with 1:6,000 dilutions of peroxidase-conjugated secondary antibodies (anti-mouse IgG [A4416, Sigma-Aldrich]; anti-rabbit IgG [A0545, Sigma-Aldrich]) for 60 min at room temperature. The blots were washed twice again with TBST, and chemiluminescent signals were visualized using a Chemi-Lumi One Super imager (Nacalai Tesque, Kyoto, Japan). Protein band intensities were assessed using ImageJ software (developed by Wayne Rasband, National Institutes of Health). Triplicate experiments yielded nearly identical results.

### Real-time reverse transcription PCR (RT-PCR) analysis

Total cell RNA, cultured in a 35-mm dish, was extracted from cells using ISOGEN II (Nippon Gene, Osaka, Japan) at 0,8 h after the start of incubation in the IH group (N0, H3) and 8 h after the start of incubation in the continuous hypoxia group (SH). Reverse transcription was performed using ReverTra Ace® qPCR RT Master Mix (Toyobo, Osaka, Japan) according to the manufacturer’s instructions. Quantitative real-time RT-PCR was performed using Applied Biosystems 7300 Real-Time PCR System (Applied Biosystems, Carlsbad, CA) with TaqMan gene expression assay (Applied Biosystems) for HIF-1α (Hs00153153_m1), SOX9 (Hs00165814_m1), ACAN (Hs00153936_m1) Adamts4 (Hs00192708_m1), and MMP13 (Hs00942584_m1). Each 20 μL reaction mixture contained 2 μL of cDNA and 10 μL TaqMan® Gene Expression PCR Master Mix (TOYOBO, Osaka, Japan) for the target gene. The amplification protocol consisted of 40 cycles of denaturation at 95°C for 15 s and annealing and extension at 60°C for 1 min. Relative changes in gene expression were determined using the comparative CT method. The 18S ribosomal RNA was used as the internal control (forward primer, 5′-ATGAGTCCACTTTAAATCCTTTAACGA-3′; reverse primer, 5′-CTTTAATATACGCTATTGGAGCTGGAA-3′; probes, 5′-[FAM]ATCCATTGGAGGGCAAGTCTGGTGC[BHQ]-3′). Each experiment was repeated twice.

### Cell viability analysis

Cell viability was determined by the RealTime-Glo™ MT Cell Viability Assay (Promega, Madison, WI) as described. Before starting culture under hypoxic culture conditions, the cells in 96-well plates were washed carefully with PBS, and 100 μl of culture medium was mixed with 0.1 μl of MT Cell Viability Substrate and NanoLuc® Enzyme and was added to each well. Relative light unit (RLU) was measured with Centro LB960 (Berthold Technologies, Bad Wildbad, Germany) after 8 h (H3, HS) of incubation under intermittent and SH. Cells cultured under normoxic conditions for 8 h were used as a control. RLU was also measured in the IH group, which underwent the same hypoxic cycle for up to 23 hours (16 hours total hypoxia, 8 hours normoxia), the SH group, which was incubated under normoxic conditions for 16 hours followed by 8 hours of hypoxia, and the control group, which was incubated under normoxic conditions for 23 hours. Each experiment was repeated twice.

### Preparation of animals

Experiments were performed on 6- and 8-week-old male Wistar rats (Shimizu Laboratory Suppliers, Kyoto, Japan). A total of 34 rats were used; no animals were excluded in this study. Animal experiments were performed with approval from the Experimental Animals Committee, Kyoto Prefectural University of Medicine (No. M2020-533).

### Rearing in a hypoxic chamber

**Fig 2:**
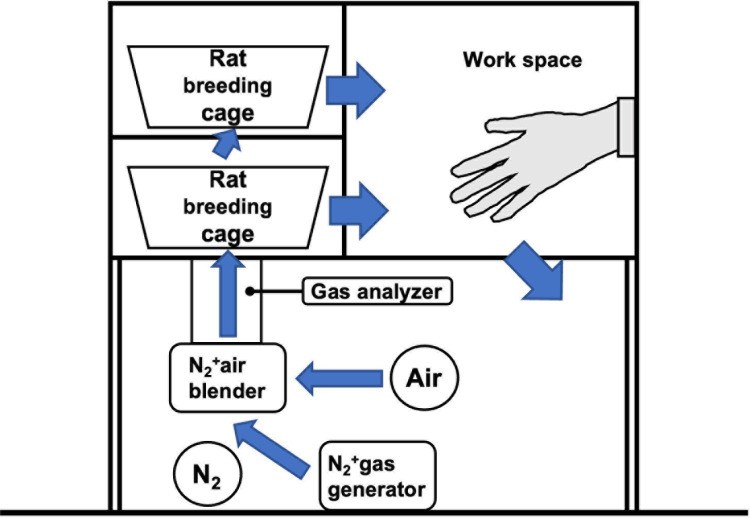
A novel hypoxia chamber was devised and fabricated. Nitrogen was generated from an N2 + gas generator and mixed with ambient air by an N2 + air blender to produce hypoxia. Oxygen concentration was measured at any location using a gas analyzer and adjusted as desired by circulating the nitrogen through the chamber.

A chamber with adjustable oxygen concentration (Natsume Seisakusho Co., Ltd., Wakenyaku Co., Ltd., Kyoto, Japan) was used to maintain a low-oxygen environment. Nitrogen generated by the N2 + gas generator was mixed with ambient air at an arbitrary ratio using an N2 + air blender to generate hypoxia and circulated to the rat breeding cage to create a hypoxic environment in the chamber. The oxygen concentration can be measured at any location using a gas analyzer and adjusted arbitrarily within the range of 4–21% by adjusting the air blender (Fig 2). In an experiment investigating hypoxic adaptation in Wistar rats, it has been reported that 38% of the animals died at an altitude of 10,000 meters, 83% died at an altitude of 10,500 meters, and a higher percentage died at an altitude of 11,000 meters than at 10,500 meters [31]. Given that 10,000 meters is equivalent to an environment with 4% oxygen, the oxygen concentration in the chamber was adjusted from 4% to 21% with reference to this literature in the present study, and the effects of the hypoxic environment on the animals were investigated preliminarily. Six-week-old male Wistar rats were continuously reared at oxygen concentrations of 4%, 6%, 7%, 9% (each n =  1), and 12% and 21% (each n =  3). The rats died after 6 h of rearing in 4% oxygen, 1 day of rearing in 6% oxygen, and 12 days of rearing in 7% and 9% oxygen. Compared to rearing at an oxygen concentration of 21%, that at 12% had a smaller effect on the general condition of the rats, including locomotion and weight loss. Accordingly, the oxygen concentration was set at 12% in subsequent experiments.

### Rat OA mode

A rat OA model was created by injecting monosodium iodoacetate (MIA; Sigma-Aldrich, St. Louis, MO) from a 27-G needle, at a volume of 1 mg in 50 µl of PBS, into the right knee joint of 8-week-old male Wistar rats that were anesthetized with a mixture of 0.375mg/kg medetomidine, 2.0mg/kg midazolam, and 2.5mg/kg butorphanol.

### Rearing in IH

The prepared rat OA model was divided into two groups: one where animals were reared in intermittent hypoxic conditions (IH group, n =  14) and one where they were reared in a steady oxygen environment (normoxia group, n =  14). In the IH group, the rat OA model was kept in a 12% hypoxic environment for 24 h and then in a steady oxygen environment for 24 h; this was repeated alternately for a total of 28 days. In the normoxia group, the rats were continuously kept in a 21% steady-state oxygen environment. All the rats were maintained in a 12-h light/dark cycle and had free access to food and water.

### Histological analysis

**Fig 3:**
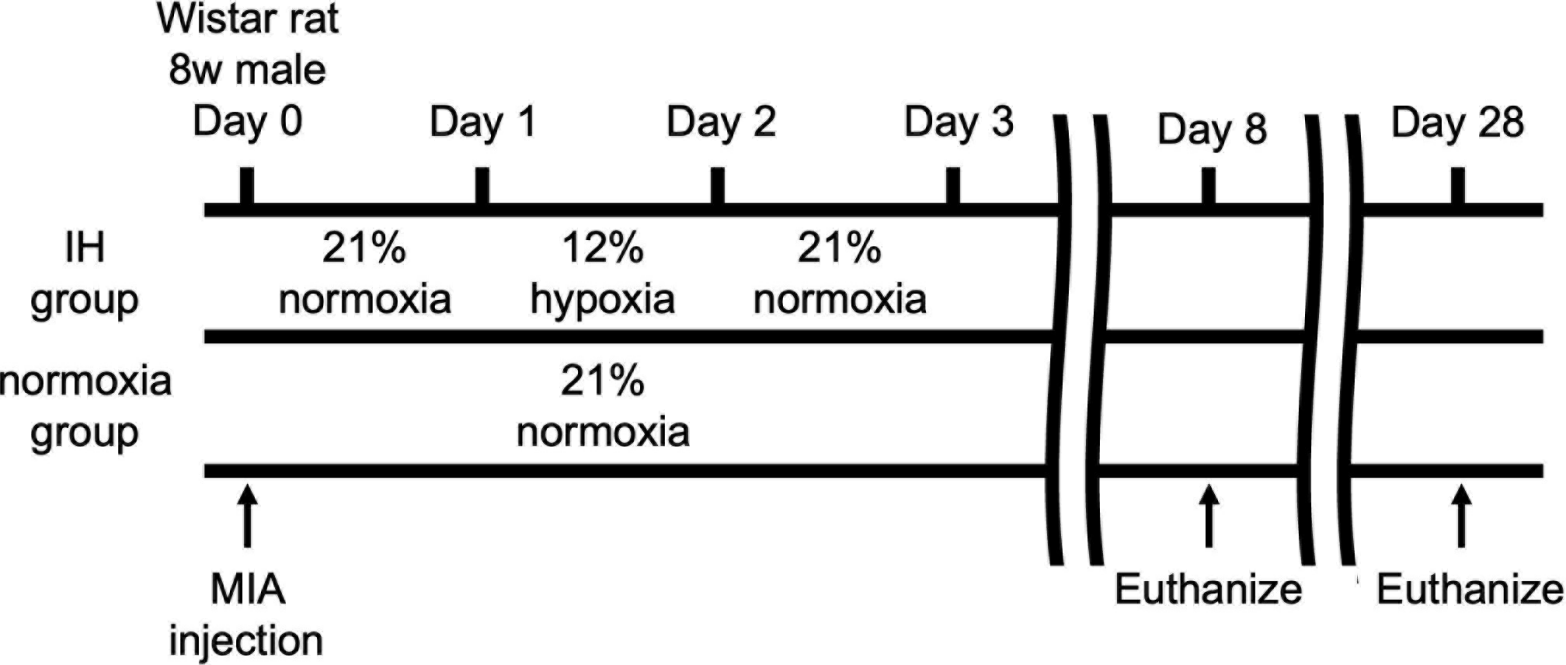
The rat OA model was created by intra-articular MIA injection into the right knees of 8-week-old male Wistar rats. Rats were divided into two groups—namely, the IH group, which included rats reared under 12% hypoxia in a hypoxic chamber every 24 h after MIA injection, and the normoxia group, which comprised rats reared under 21% normoxia (n =  14).

Rats were deeply anesthetized with pentobarbital and euthanized at 8 and 28 days after MIA injection (Fig 3). The right knee joints of the rats were removed, and the area of cartilage degeneration was detected by black ink staining; the joint was fixed with 4% paraformaldehyde (Wako, Osaka, Japan), at 25°C degreased in 95% ethanol for 3 days, demineralized with 10% EDTA for 5 weeks, and embedded in paraffin. Sagittal sections of 6 µm thickness were prepared from the medial center of the knee and stained with HE and Safranin O. Semiquantitative histopathological evaluation was performed using a modified Mankin scale [32]. For HIF-1α immunohistochemistry, paraffin-embedded sections were de-paraffinized in xylene, rehydrated through graded alcohol, and immersed. Endogenous peroxidase activity was blocked by incubating the sections in 3% H_2_O_2_ in methanol for 15 min. The sections were incubated 50 min at 25°C with rabbit polyclonal anti-HIF-1α (ab114977; abcam) at a 1:100 dilution. After extensive washing with PBS, the sections were incubated in Histofine® Simple Stain Rat MAX-PO (NICHIREI BIOSCIENCES INC., Tokyo, Japan) for 30 min at 25°C. Immunostaining was detected using DAB staining. Counter staining was performed with Mayer’s hematoxylin. The total number of chondrocytes, as well as the number of HIF-1α positive chondrocytes were determined at 40 × magnification.

### Statistical analysis

Data are expressed as mean ±  SD and were analyzed using EZR (Saitama Medical Center, Jichi Medical University, Saitama, Japan), a graphical user interface in R (The R Foundation for Statistical Computing, Vienna, Austria) [33]. The data were confirmed to be normally distributed by the Kolmogorov-Smirnov test and analyzed by t-test, Mann-Whitney’s U test, Pearson’s chi-square test and one-way analysis of variance, followed by the Tukey–Kramer test for post-hoc analysis. P <  0.05 was considered statistically significant.

## Results

### Effects of IH on protein expression in HCHs

**Fig 4:**
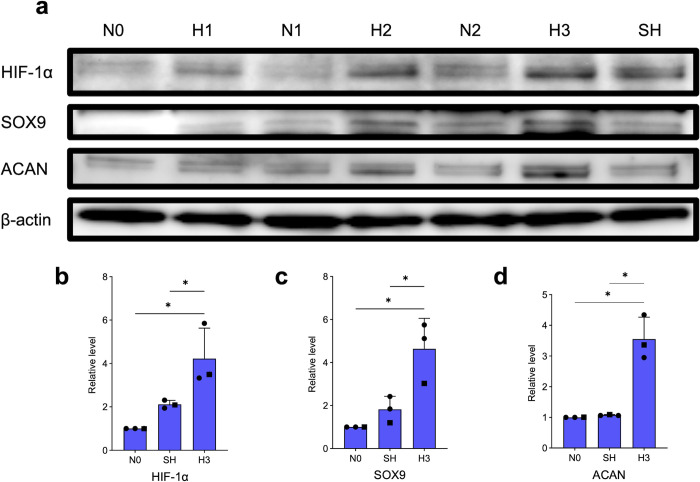
HIF-1 α, SOX9, and Aggrecan protein expression was analyzed by western blotting after HCH incubation in intermittent and continuous hypoxia. Triplicate experiments yielded nearly identical results (n =  3). Each value represents the mean ±  SD. * p <  0.05. The different symbols in the scatterplot indicate the respective donors.

The effect of intermittent hypoxic stimulation of HCHs on protein expression was investigated (Fig 4). There were no differences in the morphology of HCH in each group and no obvious abnormalities due to hypoxic culture conditions. HIF-1α expression was enhanced in H1 under the first hypoxic stimulus and decreased in N1 under steady oxygen. HIF-1α expression was further enhanced in H2 after the second hypoxic stimulation than in H1. The HIF-1α expression was also decreased in N2 under the second steady oxygen stimulus, as in N1. The third hypoxic stimulus, H3, enhanced HIF-1α expression more than H1 and H2. Protein expression of HIF-1α was enhanced after each cycle of hypoxic stimulation. Of all the experiments, the expression of H3 was highest. Protein expression of SOX9 and Aggrecan associated with chondrogenesis was similarly enhanced with repeated hypoxic stimulation in H2 and H3 compared to that in H1. Protein expression of HIF-1α, SOX9, and Aggrecan was significantly enhanced in H3 compared to that in SH with sustained hypoxic stimulation (p <  0.05).

### Effects of IH on gene expression in HCHs

**Fig 5:**
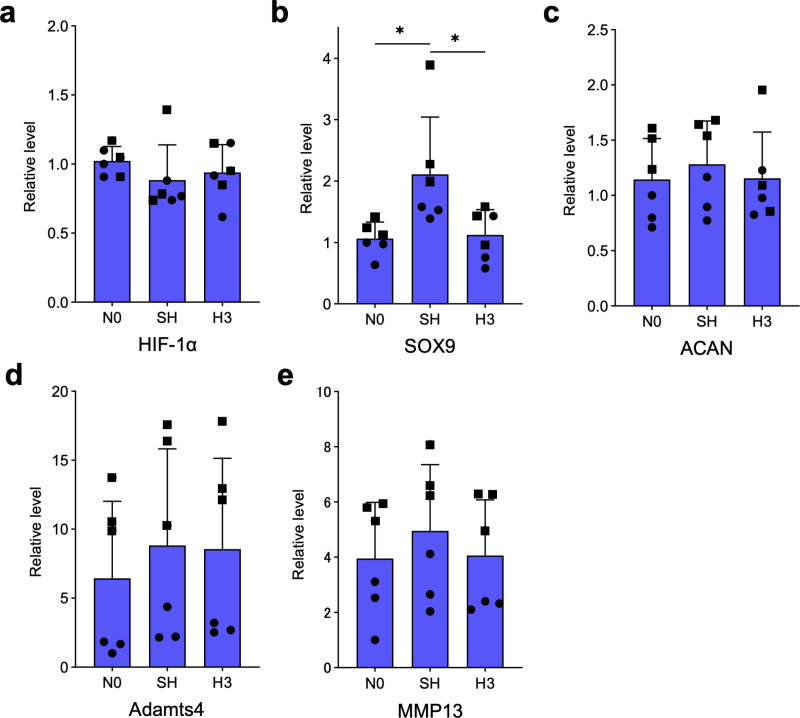
Gene expression of HIF-1 α, SOX9, Aggrecan, Adamts4, and MMP13 was analyzed by real-time RT-PCR at 0 and 8 h (N0, H3) after the start of culture in the IH group and at 8 h in the continuous hypoxia group (SH). Each experiment was repeated twice (n =  6). Each value represents the mean ±  SD. *  p <  0.05. The different symbols in the scatterplot indicate the respective donors.

We examined the effect of intermittent hypoxic stimulation of HCHs on gene expression (Fig 5). SOX9 gene expression levels in the chondrocytes of the SH group were significantly elevated compared to other groups (p <  0.05). HSP70, ACAN, Adamts4, and MMP13 gene expression levels were not significantly different.

### Effects of IH on cell viability of HCHs

**Fig 6:**
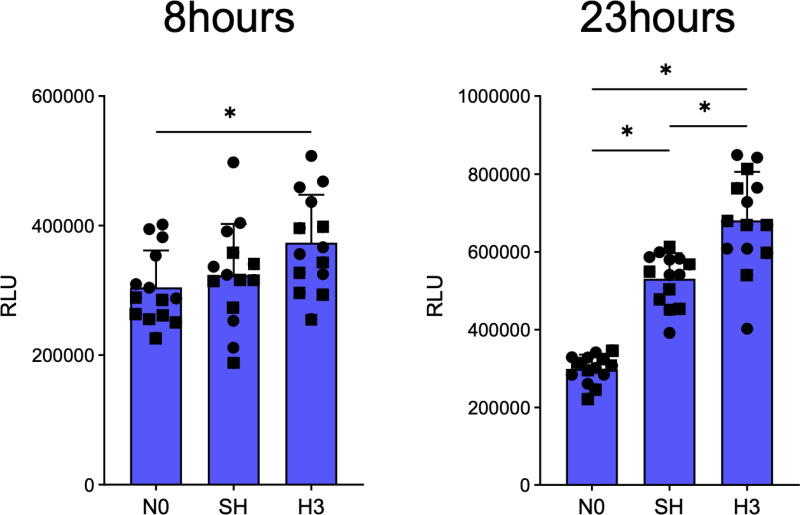
Cell viability of HCHs after 8 and 23 h of incubation in normoxia, IH, and SH was evaluated using the RealTime-Glo™ MT Cell Viability Assay. Each experiment was repeated twice (n =  14). Each value represents the mean ±  SD. *  p <  0.05. The different symbols in the scatterplot indicate the respective donors.

The effect of intermittent hypoxic stimulation on cell viability of HCHs was evaluated (Fig 6). The RLU was significantly higher (p < 0.05) in cells cultured in IH for both 8 and 23 hours of incubation than in cells cultured in normoxia or SH. Cells cultured in SH were not significantly different from cells cultured in normoxia for a total of 8 hours, but were significantly higher for a total of 23 hours.

### Effects of IH on a rat OA model in 8 days

**Fig 7:**
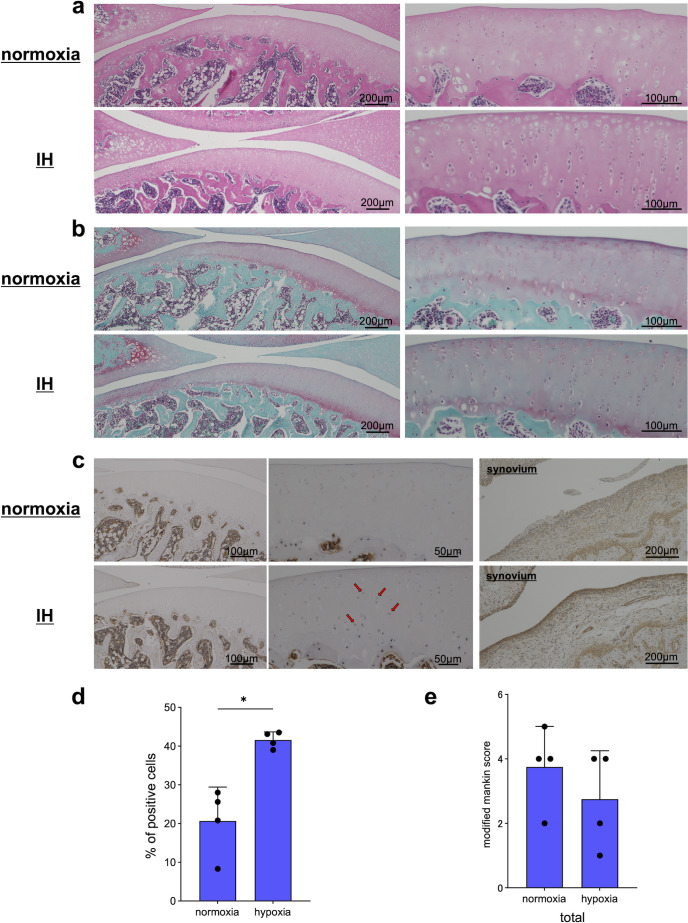
The rat OA model was reared under IH for 8 days, and the effects on articular cartilage were evaluated histologically. Representative photographs of hematoxylin and eosin (HE) (a), safranin O-stained (b), and HIF-1α immunostained sagittal sections (c) (red arrow: stain-positive cells). Percentage of cells positive for HIF-1α was measured (d), and OA grades were assessed using the modified Mankin score (mean ±  standard deviation [SD]) (e) (n =  4).

The rat OA models were reared under IH for 8 days, and the effects on articular cartilage were evaluated histologically. The irregularity of cartilage tissue in both the IH and normoxia groups was mild; however, the staining was decreased with safranin O staining (Fig 7a and 7b). HIF-1α immunostaining showed stronger staining in cartilage and synovium in the IH group, with a significantly higher percentage of HIF-1α positive chondrocytes (Fig 7c). The modified Mankin score was not significantly different between the two groups (Fig 7d).

### Effects of IH on a rat OA model in 28 days

**Fig 8:**
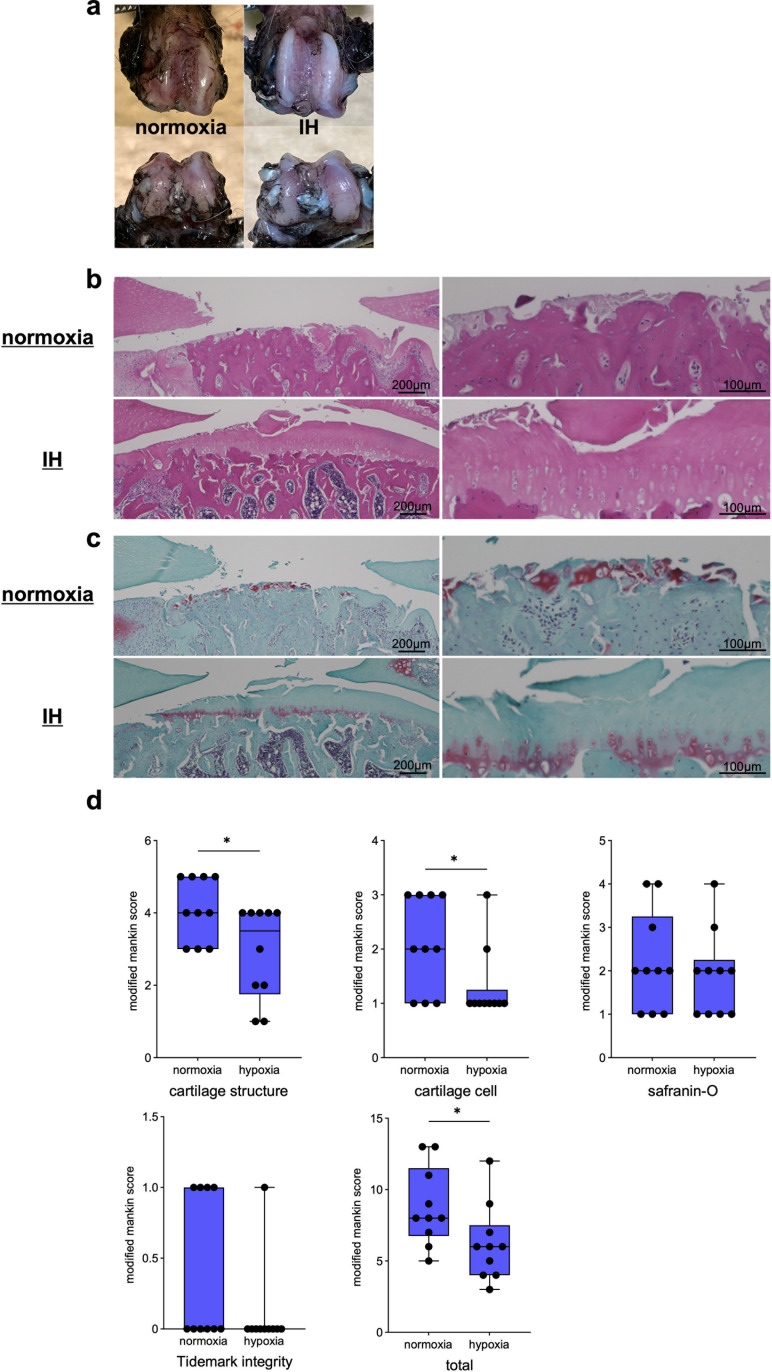
The rat OA model was reared under IH for 28 days, and the effects on articular cartilage were evaluated histologically. Representative photographs of black ink (a), HE (b), and safranin O-stained sagittal sections (c). OA grades were assessed using the modified Mankin score (mean ± SD) (d) (n =  10). * p <  0.05.

The rat OA models were reared under IH for 28 days, and the effects on articular cartilage were evaluated histologically. As in the preliminary experiment, there were no obvious complications or effects on the rat’s general conditions, such as locomotion. The normoxia group had an irregular femoral joint surface in the black ink stain; the IH group had a milder irregular femoral joint surface than the normoxia group (Fig 8a). HE staining and safranin O staining also revealed an irregular cartilage surface and thinning of the cartilage in the normoxia group, compared to the findings in the IH group (Fig 8b and 8c). The modified Mankin score showed significant improvement in the IH group (Fig 8d). Significant differences were observed, especially in the cartilage cells and structure.

## Discussion

We evaluated the effects of IH on cultured chondrocytes and a rat OA model. *In vitro*, intermittent hypoxic stimulation increased HIF-1α expression, cartilage metabolic activity, and cell viability in chondrocytes. *In vivo*, the staining of HIF-1α was stronger in IH rearing and cartilage degeneration in the rat OA model was significantly reduced compared to the steady oxygen group. Intermittent hypoxic stimulation may suppress cartilage degeneration by increasing HIF-1α expression and inhibiting degradation thereof in OA chondrocytes.

Articular cartilage maintains homeostasis by responding at the molecular level to various physiological stresses, such as mechanical stress [34,35]. As a vascularized tissue, articular cartilage is a physiologically hypoxic environment and is regularly exposed to hypoxic stress [36]. HIF-1α, a factor induced by hypoxia, acts as a positive regulator of SOX9 expression, promotes chondrocyte differentiation and matrix synthesis, is involved in autophage activation and inhibition of apoptosis, and plays an important role in the maintenance of articular cartilage homeostasis [15,37]. Synovitis increases oxygen consumption in OA, resulting in lower-than-normal oxygen levels in the joint fluid and articular cartilage and increased HIF-1α expression [38,39]. Additionally, synovitis induces angiogenesis in OA, increasing blood flow from the subchondral bone and disrupting the cartilage matrix, further increasing oxygen levels and HIF-1α expression [20,40]. It is possible that HIF-1α found in OA cartilage may be induced by other factors and function as a secondary protective factor against cartilage degeneration. Gelse et al. also found that OA progresses with HIF inhibitors in an animal model of OA and that HIF-1α expression is enhanced in OA to inhibit cartilage degeneration [41]. Therefore, basic research has been conducted to target HIF-1α for OA treatment, and it has been reported that HIF-1α can exert chondroprotective effects if it is efficiently expressed in OA. Enhanced HIF-1α expression in chondrocytes by platelet-rich plasma (PRP), dimethyloxalylglycine (DMOG), desferrioxamine (DFX), and cobalt chloride (CoCl_2_) enhances the gene expression of type II collagen and Aggrecan and exerts a cartilage protective effect [42,43]. Treatment of OA model mice with DMOG or MgCl_2_ enhances HIF1α expression and suppresses cartilage degeneration [44,45]. Induction of HIF1α by Bmal1, a clock gene, in mouse OA chondrocytes suppresses apoptosis [46]. HIF-1α enhances cell viability by inducing the expression of heat shock protein 70 and protecting chondrocytes from apoptosis [29]. HIF-1α-deficient chondrocytes have reduced cell viability due to enhanced apoptosis induced by catabolic stress [39]. Recently, it has been shown that microRNAs LncHIFCA may also regulate apoptosis via HIF-1α [47]. Thus, studies have been conducted into using drugs and other methods to stabilize HIF-1α for the prevention and control of OA progression. However, these have problems such as the lack of knowledge of their *in vivo* use and the high cost. Therefore, we focused on the regulation of HIF-1α in the hypoxic environment. Kaihara et al. reported that brief hypoxic stimulation of synovial cells increased HIF1-α, while prolonged stimulation conversely decreased HIF1-α [23]. HIF-1α has been associated with many diseases, including cancer and cardiovascular and inflammatory diseases, and has been studied in a wide variety of cells [48–50]. Additionally, studies using cancer cells and vascular endothelial cells have reported that intermittent hypoxic stimulation of cells gradually enhanced HIF-1α expression with each cycle of hypoxia, and HIF-1α was expressed more strongly than it was with sustained hypoxic stimulation [24–26]. In the present study, HIF-1α protein expression was also increased after brief hypoxic stimulation of HCCs and decreased upon reoxygenation. Each time this cycle was repeated, the HIF-1α expression increased stepwise after hypoxic stimulation, and the use of an intermittent hypoxic environment can enhance the protein expression of HIF-1α *in vitro*. Similarly, SOX9 and Aggrecan, associated with chondrogenesis, increased intermittent hypoxic stimulation protein expression more than sustained hypoxic stimulation. Compared to the culture in a steady oxygen environment, the cell viability of chondrocytes in the continuous hypoxic environment increased significantly with increasing hypoxic culture time. Conversely, the cell viability of chondrocytes was significantly increased in intermittent hypoxia, even after a short period of hypoxia. In chondrocytes, intermittent hypoxic stimulation may increase HIF-1α expression, activating cartilage metabolism and cell viability more efficiently than continuous hypoxic stimulation.

In contrast to the increase in protein expression, there was no increase in gene expression of HIF-1α, SOX9, ACAN, Adamts4, and MMP13 in the IH group compared to the steady-state oxygen and SH groups. In experiments using adenocarcinoma A549 cells, intermittent hypoxic culture increased protein expression, but gene expression was not affected by HIF-1α and some related genes, suggesting that various oxygenation conditions may directly affect these factors at the protein level [51]. It is also possible that there are constituents that directly affect these factors at the protein level in chondrocytes. In addition, this study was conducted in *in vitro* experiments in monolayer cultures that had not been stimulated with interleukins or other inflammatory agents, which may differ from the actual behavior of chondrocytes; however, the detailed mechanism has not been elucidated at this time.

In this study, arthropathy was induced by intra-articular injection of MIA, a widely used method to create animal OA models; MIA induces intra-articular inflammation with synovitis, resulting in cartilage degeneration include reduction of proteoglycans, inhibition of chondrocyte proliferation, and induction of chondrocyte death [52]. As synovitis is thought to cause changes in the oxygen environment within the joints of OA, mainly due to synovitis, the present study experimented with an MIA model in which OA is induced by synovial inflammation. Our MIA model rats, reared under a steady oxygen environment, showed a decrease in safranin O staining at 8 days, and thinning of the cartilage tissue and a marked decrease in staining of safranin O at 28 days. Additionally, when rat OA models were kept in an intermittent hypoxic environment every 24 h, compared to the steady oxygen group at 8 days, the extent of OA was not significantly different between the steady oxygen group, but HIF-1α immunostaining showed stronger staining in both the articular cartilage and synovium in the IH group. Furthermore, at 28 days, the IH group showed mild destruction of cartilage structures and significantly reduced degeneration of the articular cartilage. For cartilage degeneration caused by MIA, Moon et al. found that shifting extracellular matrix homeostasis in anabolic direction reduces pain and articular cartilage destruction caused by OA [53]. Cifuentes et al. also found that increased expression of proteoglycans by moderate exercise load can inhibit the destruction of articular cartilage in response to cartilage degeneration caused by MIA [54]. Our results suggest that an intermittent hypoxic environment efficiently expresses HIF-1α in chondrocytes, causing an increase in cartilage metabolism and suppressing cartilage degeneration in rat OA models. HIF-1α expression is also observed in the nucleus pulposus cells of the intervertebral disc, which, like articular cartilage, is composed of cartilage components. Although the degeneration mechanisms of intervertebral discs and articular cartilage differ in certain respects [55], and the patterns of HIF-1α expression are also different [56], HIF-1α expression also suppresses intervertebral disc degeneration [57]. Therefore, the present findings may be applied to analysis in this field.

This study has several limitations. First, the mechanism by which HIF-1α is increased by IH is unknown. The mechanisms by which repeated hypoxic stimulation increases HIF-1α have been the subject of various theories; Martinive et al. reported that, in vascular endothelial cells, activation of the PI3K/Akt pathway during reoxygenation contributes to increased HIF-1α expression during subsequent hypoxic stimulation [58]. Toffoli et al. stated that the continuous increase in PKA activity due to IH cycles may promote the phosphorylation of HIF-1α and increase its transcriptional activity [59]. It is also known that more reactive oxygen species (ROS) are produced during reoxygenation than during hypoxia [60]. Malec et al. reported that ROS-enhanced production by IH may be associated with increased expression of the antioxidant enzyme regulator nuclear factor erythroid 2-related factor 2 (NRF2), which induces HIF-1α stabilization [51]. Second, the MIA model was used as an animal OA model in this study, and the effects of hypoxia in models where OA is induced by mechanical stress, such as ACLT, are unknown.

## Conclusions

This study is the first to demonstrate the chondroprotective effect of an intermittent hypoxic environment on articular cartilage. If HIF-1α can be efficiently regulated via intermittent hypoxic environment exposure, it may become a new effective method for prevention and treatment for OA patients. The control of HIF-1α using a hypoxic environment is inexpensive and can be easily introduced in an outpatient clinic or combined with existing exercise therapy. Furthermore, biomaterials technology, which has been studied in recent years [61–63], may be even more effective when performed in a hypoxic environment.

## Supporting information

S1 File The values used to build graphs. (XLSX)

S2 File **S1 Fig**. Original image for blot of HIF-1α. **S2 Fig**. Original image for blot of SOX9. **S3 Fig**. Original image for blot of ACAN. **S4 Fig**. Original image for blot of β-actin.
(DOCX)
