# Supplementary material for: Intermittent hypoxic stimulation promotes efficient expression of Hypoxia-inducible factor-1α and exerts a chondroprotective effect in an animal osteoarthritis model
Source: PLoS One. 2025 Apr 1;20(4):e0319976. doi: 10.1371/journal.pone.0319976 (PMC11960973; doi:10.1371/journal.pone.0319976)
Supplement: S2 File — S1 Fig. Original image for blot of HIF-1α. S2 Fig. Original image for blot of SOX9. S3 Fig. Original image for blot of ACAN. S4 Fig. Original image for blot of β-actin. (DOCX) [file pone.0319976.s002.docx]

**Supporting figures**

**S1 Fig.**

**
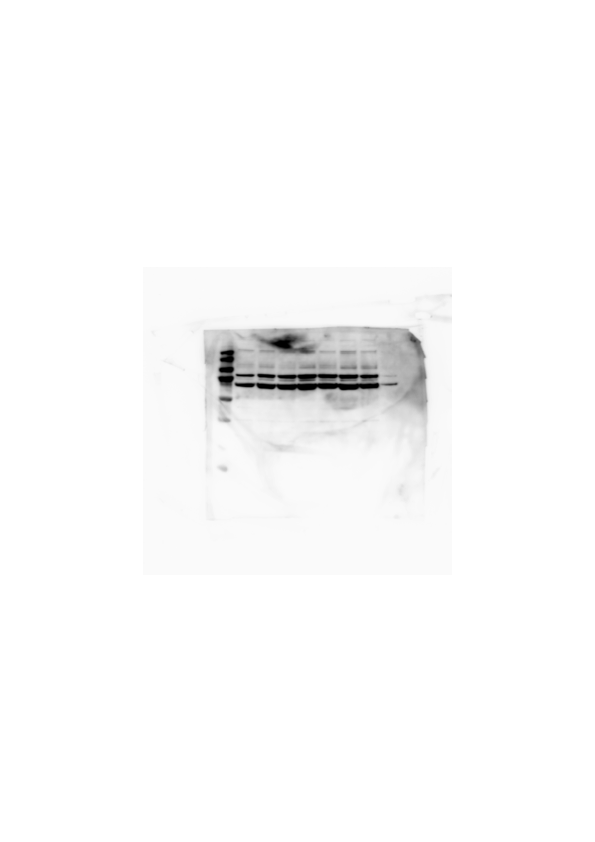
**

**S1 Fig.　 Original image for blot of HIF-1α.**Red arrow indicate HIF-1α band positions.

**S2 Fig.**

**
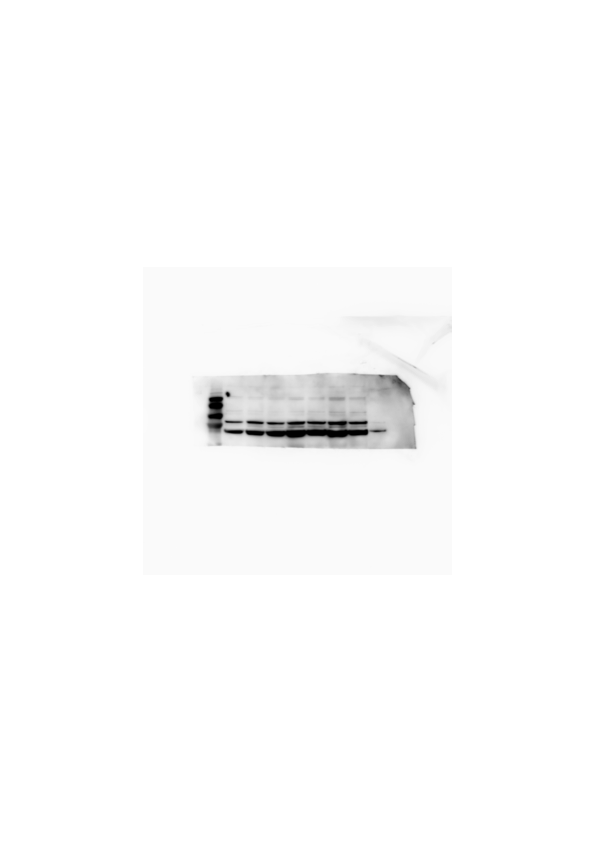
**

**S2 Fig.　 Original image for blot of SOX9.**Red arrow indicate SOX9 band positions.

**S3 Fig.**

**
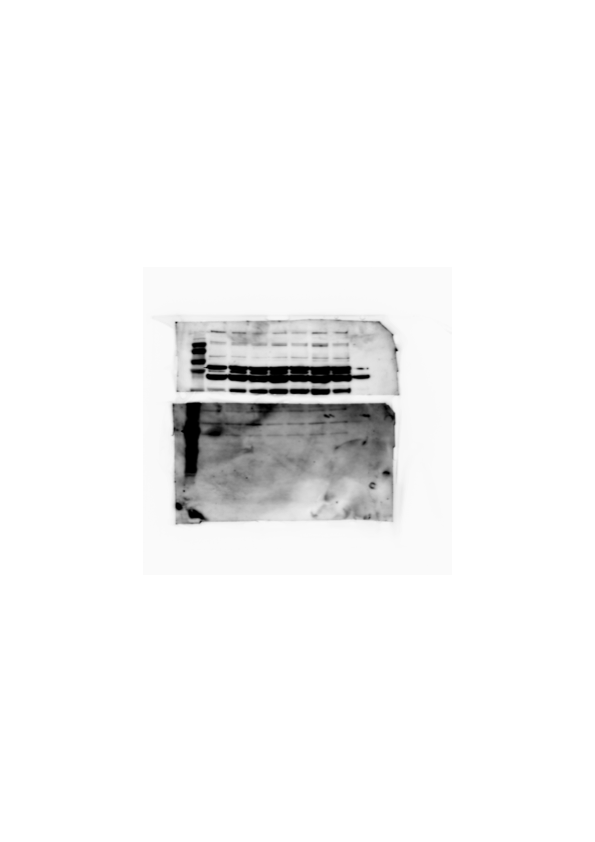
**

**S3 Fig.　 Original image for blot of ACAN.**Red arrow indicate ACAN band positions.

**S4 Fig.**

**
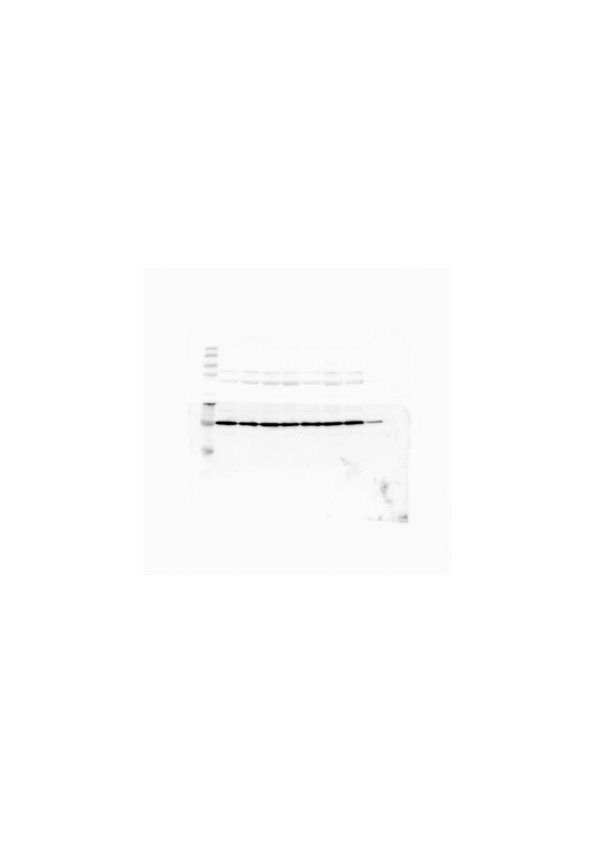
**

**S4 Fig.　 Original image for blot of β-actin.**Red arrow indicate β-actin band positions.
